# Multithreaded comparative RNA secondary structure prediction using stochastic context-free grammars

**DOI:** 10.1186/1471-2105-12-103

**Published:** 2011-04-18

**Authors:** Zsuzsanna Sükösd, Bjarne Knudsen, Morten Værum, Jørgen Kjems, Ebbe S Andersen

**Affiliations:** 1 Interdisciplinary Nanoscience Center, Aarhus University, Aarhus, Denmark; 2 Department of Molecular Biology, Aarhus University, Aarhus, Denmark; 3 CLC bio, Aarhus, Denmark

## Abstract

**Background:**

The prediction of the structure of large RNAs remains a particular challenge in bioinformatics, due to the computational complexity and low levels of accuracy of state-of-the-art algorithms. The *pfold *model couples a stochastic context-free grammar to phylogenetic analysis for a high accuracy in predictions, but the time complexity of the algorithm and underflow errors have prevented its use for long alignments. Here we present *PPfold*, a multithreaded version of *pfold*, which is capable of predicting the structure of large RNA alignments accurately on practical timescales.

**Results:**

We have distributed both the phylogenetic calculations and the inside-outside algorithm in *PPfold*, resulting in a significant reduction of runtime on multicore machines. We have addressed the floating-point underflow problems of *pfold *by implementing an extended-exponent datatype, enabling *PPfold *to be used for large-scale RNA structure predictions. We have also improved the user interface and portability: alongside standalone executable and Java source code of the program, *PPfold *is also available as a free plugin to the CLC Workbenches. We have evaluated the accuracy of *PPfold *using BRaliBase I tests, and demonstrated its practical use by predicting the secondary structure of an alignment of 24 complete HIV-1 genomes in 65 minutes on an 8-core machine and identifying several known structural elements in the prediction.

**Conclusions:**

*PPfold *is the first parallelized comparative RNA structure prediction algorithm to date. Based on the *pfold *model, *PPfold *is capable of fast, high-quality predictions of large RNA secondary structures, such as the genomes of RNA viruses or long genomic transcripts. The techniques used in the parallelization of this algorithm may be of general applicability to other bioinformatics algorithms.

## Background

Recent years have seen an explosion in the amount of biological data available from large-scale genome sequencing projects, but this increase has not been met by a corresponding increase in single-core computer power to bioinformatically analyze this data. It is therefore predicted that the scientific community will face serious computational problems in the coming years in their efforts to interpret genomic data. The prediction of RNA secondary structure remains a particularly challenging problem, in a large part due to its computational complexity: even without pseudoknot prediction, the execution time of state-of-the-art algorithms scales as O(*L*^3^) or worse with the length of the sequence, *L*. One way to address this problem is by exploiting heuristics to reduce complexity, but this happens at the cost of accuracy in predictions, which is particularly detrimental in the case of already inaccurate algorithms. Another possibility is to apply emerging multithreading paradigms to more accurate algorithms, and obtain the precise results in a fraction of the time.

RNA secondary structure prediction algorithms are typically based on either thermodynamic or stochastic context-free grammar (SCFG) models, and are implemented using dynamic programming. A recent review [1] gives an overview over existing tools. Previous attempts to parallelize RNA structure prediction algorithms have included thermodynamic prediction [2-5] and the SCFG-based profiling of RNAs [6], as well as massively parallel genetic algorithms [7] and hardware-accelerated folding on FPGA chips [8] and GPUs [9]. Despite improved runtimes, the accuracy of these algorithms remains low, due to models that may not be appropriate for very long sequences.

**Figure 1:**
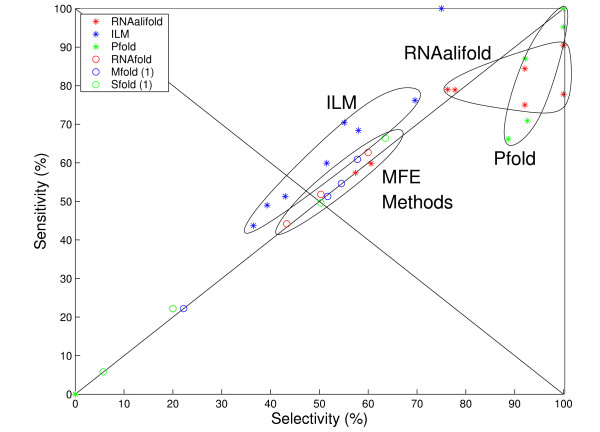
**Comparison of RNA secondary structure prediction algorithms**. The receiver operating characteristic (ROC) plots obtained from an independent study [12] simultaneously display both sensitivity and selectivity for various RNA secondary structure prediction algorithms, including pfold, iterated loop matching (ILE) and RNalifold. Accuracies of the minimum free energy (MFE) methods (MFold, RNAFold and SFold) are shown to provide a base-line. Points on the line *X *= *Y *are as sensitive as they are selective, points below this line indicates a greater selectivity, points above indicate greater sensitivity. Points in the top right corner are "perfect" predictions. Where the variance is sufficiently small, these have been indicated with a closed curve. *Pfold *compares very favourably to other algorithms for short sequences, but for long sequences underflow errors cause both the sensitivity and the specificity to drop to zero. Figure adapted with permission from [12].

Here we focus on *pfold *[10,11], which couples a phylogenetic model to a SCFG to accurately predict the consensus structure of RNA alignments in O(*L*^3^) time [12] (Figure 1). Due to the combined approach, the *pfold *model is theoretically capable of obtaining high-quality predictions of large and biologically significant RNA structures, such as the genomes of RNA viruses. However, it has not been possible in practice to use *pfold *for such predictions: it is single-threaded, so it can take days to fold a long alignment, and it fails to predict large structures correctly due to floating-point underflow errors [12].

In this study, we address both of these issues, and create *PPfold*, an improved and multithreaded version of *pfold*. To our knowledge, *PPfold *is the first example of a multithreaded comparative RNA secondary structure prediction algorithm. We demonstrate its practical use by predicting the secondary structure of an alignment of 24 HIV-1 genomes.

## Results and Discussion

### Algorithm

*PPfold *uses the same combined evolutionary and SCFG model as *pfold *[10,11]. A summary of this model is provided in the Methods section. Here we focus strictly on the parts of *PPfold *that present improvements on *pfold*.

#### Multithreading the phylogenetic calculations

After estimating the phylogenetic tree, the *pfold *algorithm calculates column- and column-pair likelihoods, based on post-order traversal through the tree. We have only distributed the calculation of column-pair likelihoods, as this is the most time-consuming part with a time complexity of O(*L*^2^), where *L *is the length of the alignment. It is desirable to distribute the calculations as evenly as possible, so all processing units have an equal workload. As all column-pairs are treated independently, a natural division for multithreading arises by grouping a number of column pairs together in such a way that there are as many groups with equal numbers of column pairs as processing units. However, a unique mapping from the number of groups, *n*, to the size of each group, *s*, does not exist.

Column-pair likelihoods are symmetric, so in total there are  column pairs to calculate. For simplicity, we chose to distribute these on the basis of the first iterator column: to each group, we incrementally assign as many first iterator columns (and all their pairing columns) as possible, such that the total number of column pairs in all groups up to group number *k *> 0 does not exceed

**Figure 2:**
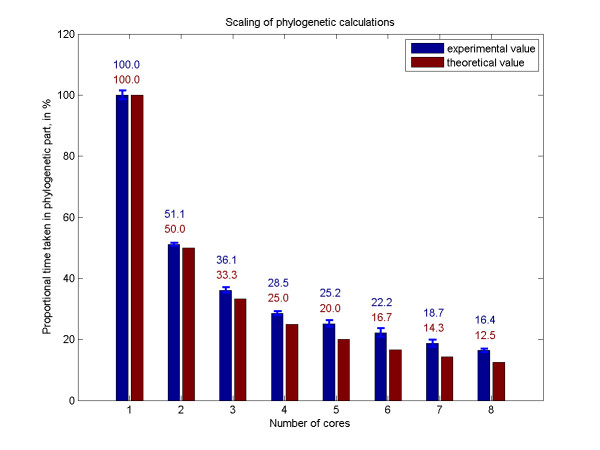
**Reduction in runtime for the phylogenetic part of the algorithm**. The execution time of the phylogenetic part of the algorithm is reduced proportionally to the number of cores. We used 40 divisions for the folding of 30 × 3000 nt, on a Intel(R) Xeon(R) E5420 CPU, 8 cores, 2.50 GHz, 32 GB RAM, and enabled different number of cores to be used by PPfold by varying the size of the threadpool. Here we are plotting the mean and standard deviation of 4 measurement points, scaled as a fraction of one-core runtime.

This provides an approximately even distribution of workload to the processing units, and we observe a corresponding reduction in running time on multicore machines. (Figure 2)

#### Multithreading the inside-outside algorithm

The inside-outside algorithm fills two lower triangular matrices of dimension *L *for each nonterminal symbol of the grammar, through dynamic programming. The Knudsen-Hein grammar contains 3 nonterminal variables, so in total there are 6 such matrices to be filled. The algorithm exhibits heavy dependencies, making its distribution into independent "jobs" nontrivial. In *PPfold*, we have chosen an asynchronous wavefront computational approach that exploits the geometry of the algorithm.

**Figure 3:**
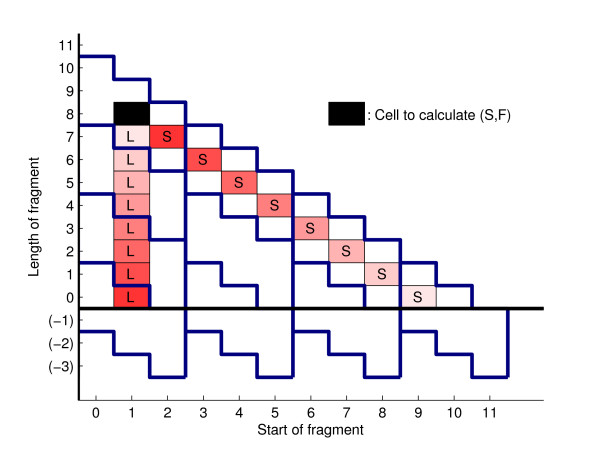
**Division of calculations in the inside-outside algorithm**. In order to apply the bifurcation rules of the grammar to the cell marked in black, the inside algorithm requires the values in the red cells to be known (from nonterminals L (vertical) and S (diagonal)). The products of values in cells of the same shade of red are added to the value in the black cell. Thick blue lines illustrate the defined sector boundaries, for a choice of divisions where the dimension of each sector is 3. All sectors have the same size; points that lie outside the region to be calculated (for example, negative subsequence lengths) are stored but ignored in the calculations.

**Figure 4:**
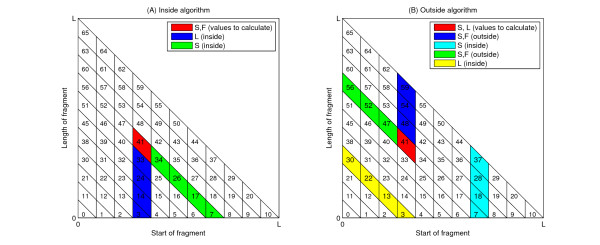
**Dependencies of sectors in the inside-outside algorithm**. The geometry for job divisions is inspired by the dependency structure of the (a) inside and (b) outside algorithms. For illustration purposes, here the number of divisions of the sequence is 11, giving rise to 66 sectors in total. (Sectors are numbered starting from 0, in accordance with the algorithm implementation.) In order to calculate values in the sector marked red, the indicated values in the coloured sectors must be known.

**Figure 5:**
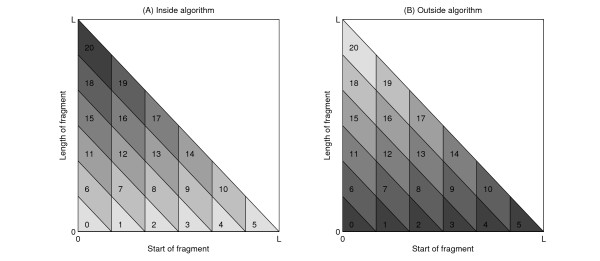
**Workload of jobs in the inside-outside algorithm**. The execution time is proportional to job height, as illustrated here for the (a) inside and (b) outside algorithms. Jobs executed last take longest (top job in inside algorithm, bottom row in outside algorithm). (The expectation value calculations are analogous to the inside algorithm, but each job takes comparatively shorter time.) The linear dependence on job height gives rise to the O(*n*^3^) time complexity.

We divide the triangle into equal-sized parallelogram-shaped "sectors" (Figure 3). We will refer to the number of sectors in the first row of the triangle by *N*. The dependency of the sectors on each other in the inside and outside parts of the algorithm is illustrated by Figure 4; the values for all nonterminals in each sector can be evaluated once all dependencies are completed. A "job" thus entails the evaluation of either the inside or the outside variables corresponding to a sector for all nonterminal variables in the grammar. The workload in jobs is not equally distributed, as illustrated by Figure 5.

By design, jobs are self-sufficient objects created only when their dependencies are completed: they contain all data necessary for the calculation of the values in the corresponding sector in order to also allow distribution to a non-shared memory framework. Asynchronous implementation makes it possible to execute jobs immediately after the necessary dependencies are completed, rather than waiting for all jobs in the same row to complete. (Additional File 1) This is ideal for a setting where executor units have different capabilities, such as a grid of personal computers.

**Figure 6:**
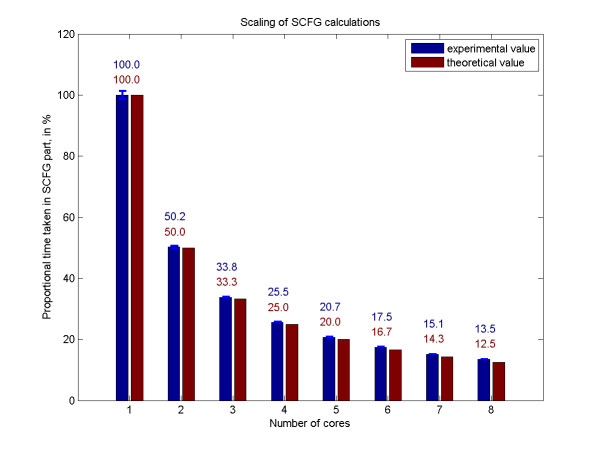
**Execution time of SCFG part on multicore machines**. The execution time of the SCFG part of the algorithm is reduced proportionally to the number of cores for a sufficiently high number of divisions. We used 40 divisions for the folding of 30 × 3000 nt, on Intel(R) Xeon(R) E5420 CPU, 8 cores, 2.50 GHz, 32 GB RAM, and enabled different number of cores to be used by PPfold by varying the size of the thread pool. Here we are plotting the mean and standard deviation of 4 measurement points, scaled as a fraction of one-core runtime.

It is important to note that multithreading is not possible for all parts of the algorithm: for example, the job at the top of the triangular matrix has to be executed by one processing unit without any simultaneous calculations. Therefore it is ideal to choose *N *>>*u*, where *u *is the number of available processing units. In the limit *N *→ ∞, the theoretical execution time on *u *processing units is reduced to  of the execution time on one processing unit, and this is also what we observe in practice. (Figure 6) We note that this method of divisions is generally applicable to any bifurcating SCFG, and thus may be used for the parallelization of other algorithms also.

Memory use is optimized with a large number of divisions, where only the lower triangular matrices are stored. However, the space complexity of the algorithm remains O(*L*^2^), and roughly 6 GB of memory are needed to fold a 10000 nucleotide-long alignment.

#### Multithreading expectation maximization

In contrast to many SCFG-based RNA secondary structure prediction programs, *pfold *returns the structure with the maximum number of expected correctly predicted nested structural elements, instead of the maximum likelihood estimate. To find this structure, it is necessary to calculate a matrix of expectation values for every fraction of the alignment, similarly to the inside algorithm. The details of this are described in the Methods section. As these calculations also contain bifurcations, they are distributed analogously to the inside algorithm.

#### Underflow

Floating-point underflow arises commonly in dynamic programming for the folding of long RNAs, due to the multiplication of several thousands of low probabilities with each other. It has effectively prevented the correct folding of large RNA alignments with *pfold*, as the values of the inside-outside variables decrease both with the length of alignment the number of sequences in it. A common solution is to calculate with log-probabilities, implementing addition as a "log sum" function with a lookup table. Other possible solutions include multiplying the rules of the grammar with a factor, such that underflow is reached more slowly, thus extending the foldable length of alignments, or multiplying a block of probabilities lower than a certain cutoff value by a scaling factor.

In *PPfold *we have taken an alternative approach and implemented an extended exponent datatype, consisting of a float "fraction" and an integer "exponent" (base 2) part. Together, 64 bits are used to store each number - the same amount of space as a double-precision floating point number, so the overall memory requirements of the algorithm are not increased substantially. For each nonterminal symbol (in the inside, outside and expectation parts of the algorithm), we store two 2-dimensional arrays: one for the exponents and one for the fractions. For every operation, we combine numbers from these arrays using custom bitmasking and bitshifting methods.

### Implementation

PPfold has been written in Java 5.0, and integrated into the CLC Workbenches using the CLC Developer Kit (version 3.31) API. The source code and executables are available for download at http://www.daimi.au.dk/~compbio/pfold/downloads.html. *PPfold *consists of an "algorithm" package that can be compiled and run independently of the CLC Workbenches, and a "plugin" package that provides interfacing with the CLC Workbenches.

The "algorithm" package includes all classes that are involved in the processing of sequences and alignments and creating the final structures. It has no dependencies on any CLC classes, is capable of taking command-line arguments and provides a simple graphical user interface for the selection of input files. Export formats currently supported by *PPfold *include. ct, .seq (with reliability scores) and white text, as well as basepairing probability data.

The "plugin" package makes use of the CLC Developer Kit API such that PPfold becomes a full-featured plugin to the CLC Workbenches. Futhermore, integration into "minigrid-enabled" CLC Workbenches makes it possible to distribute calculations to a collaborative mini-grid of computers [13]. The details of this aspect of our work will be published elsewhere.

### Testing and benchmarking

#### Performance

We have evaluated the speed of our algorithm for alignments of various sizes, with a varying number of cores and divisions of calculations. (Table 1) The algorithm is fast, scales well with the number of cores and makes the folding of long alignments practically possible.

**Table 1:** Performance

Alignment	divisions	1 core (sec)	2 cores (sec)	4 cores (sec)	8 cores (sec)	*pfold *(sec)
	1	5.41	4.85	4.84	4.84	
2 × 500 nt	4	5.74	3.06	2.15	2.16	0.59
	35	3.70	2.05	1.25	0.92	

	1	51.8	52.0	51.1	50.4	
20 × 1000 nt	4	46.6	27.9	19.0	19.6	7.3
	35	35.7	18.3	9.7	5.8	

	1	1738	1640	1581	1464	
30 × 3000 nt	4	1476	878	642	632	368
	35	842	424	217	123	

The actual execution time of *PPfold *(including both the phylogenetic and SCFG parts) on a Dell Precision T7500 Workstation with Dual Quad Core Intel^®^Xeon^®^X5667 3.07 GHz CPU, 24 GB RAM, is shown, for alignments of different lengths, choosing different divisions, and enabling different number of cores to be used by PPfold by varying the size of the thread pool. A small number of divisions can in some circumstances result in disproportionately long runtimes, due to the higher number of extra (unnecessary) points that are present in the calculations. The algorithm is intended to be run using a high number of divisions on all architectures. For comparison, we also include the runtimes of the original *pfold *implementation (written in C), which suffers from underflow, making the results unreliable for alignments of these lengths.

#### Accuracy

We have replicated the BRaliBase I benchmarking tests [12]. *PPfold *performs as well as *pfold *for short sequences (tRNA, RNaseP), and significantly better than *pfold *for longer sequences (SSU, LSU), as it does not suffer from the underflow problem. (Table 2)

**Table 2:** BRaliBase accuracy

Sequence	Program	Ref. basepairs	Pred. basepairs	Sensitivity, %	Selectivity, %	Correlation, %
tRNA (M)	PPfold	20	21	100.0	100.0	100.0

tRNA (M)	pfold	20	21	100.0	100.0	100.0

tRNA (H)	PPfold	20	21	100.0	100.0	100.0

tRNA (H)	pfold	20	21	100.0	100.0	100.0

RNaseP (M)	PPfold	110	110	86.4	96.0	91.2

RNaseP (M)	pfold	110	110	86.4	96.0	91.2

RNaseP (H)	PPfold	110	69	43.6	71.6	57.6

RNaseP (H)	pfold	110	69	43.6	71.6	57.6

SSU (M)	PPfold	468	420	74.4	86.1	80.2

SSU (M)	pfold	468	0	0.0	0.0	0.0

SSU (H)	PPfold	468	373	68.4	89.1	78.8

SSU (H)	pfold	468	373	68.4	89.1	78.8

LSU (M)	PPfold	839	830	58.2	62.5	60.3

LSU (M)	pfold	839	0	0.0	0.0	0.0

LSU (H)	PPfold	839	754	52.2	61.0	56.6

LSU (H)	pfold	839	0	0.0	0.0	0.0

The results of our tests on the BRaliBase I dataset, using the comparison script available at http://projects.binf.ku.dk/pgardner/bralibase/bralibase1.html, are shown, both for *pfold *and *PPfold*. We carried out stem-extension separately on the *PPfold *predictions, as it is not implemented in *PPfold *at present. M: medium-similarity, H: high-similarity

#### Folding of the HIV-1 genome

**Figure 7:**
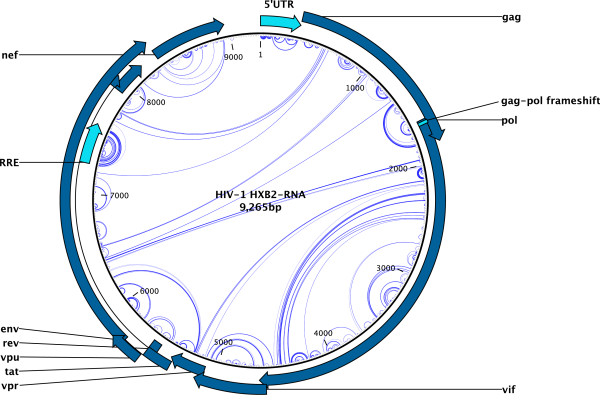
**The secondary structure of 24 HIV-1 genomes**. An overlay of an RNA structure arcplot of the *PPfold *predictions and a circularly drawn, annotated HIV-1 genome drawing made in the CLC Main Workbench is shown. *PPfold *has predicted a number of known structures on the basis of a non-adjusted sequence alignment. The arcplot was made with the "circular Feynman" diagram option of JViz.RNA [17].

To demonstrate the speed and accuracy of our algorithm, we have folded an alignment of 24 full HIV-1 genomes using *PPfold *in 65 minutes on a Dell Precision T7500 Workstation with Dual Quad Core Intel^®^Xeon^®^X5667 3.07 GHz CPU, using 6 GB of memory. In addition to predicting the consensus structure within a practically reasonable timeframe, *PPfold *has also predicted a number of known RNA structures, including the TAR, poly(A), PBS, DIS, AUG hairpins, the gag-pol frameshift and the RRE region. (Figure 7) [14] The prediction of the full consensus secondary structure of a large viral genome alignment, including phylogenetically supported long-distance interactions, has not previously been possible on practical timescales without the need for specialized hardware.

#### Numerical stability

In *PPfold*, we have reduced the representation of significant digits to 23 bits (from 52 bits in *pfold*). To assess potential numerical errors arising from this, we have folded a large HIV alignment with different divisions, such that the same computations were carried out in a different order each time. Our results show that the inside-outside variables are correct to at least 3 significant digits for a 9840-nt long alignment of 30 sequences, which we do not consider to be significant. (Table 3)

**Table 3:** Numerical stability


***Number of divisions***	***P *(*S *→ *C*_1_...*C_n_*)**

20	1.7616063 × 2^-148446^

35	1.7617458 × 2^-148446^

60	1.7618935 × 2^-148446^

The value of the S variable from the inside algorithm representing a full-length HIV-1 genome alignment (*P *(*S *→ *C*_1_...*C_n_*)), stays stable correct to at least 3 significant digits, when carrying out the calculations in a different order (with different numbers of divisions).

## Conclusions

*PPfold *is a new, multithreaded version of the *pfold *algorithm, capable of obtaining high-quality, phylogenetically supported structures for large RNA alignments in a practically reasonable time, which has not been possible previously. *PPfold *distributes both the phylogenetic and the inside-outside calculations of *pfold*, and our tests show that its speed of execution scales well with the number of executing cores. Using *PPfold*, we have been able to obtain a high-quality prediction for an alignment of 30 full HIV-1 genomes in 65 minutes on an 8-core computer. We anticipate that our algorithm will be used also for the prediction of other long RNA alignments, such as viral genomes and genomic transcripts.

## Methods

### Summary of the pfold model

Given an alignment, *pfold *creates a phylogenetic tree by neighbour-joining, then optimizes branch lengths using a maximum likelihood approach based on a general reversible evolutionary model described by Felsenstein [15]. Column-based likelihoods for unpaired nucleotides and basepairs are then obtained using post-order traversal through this tree. The phylogenetic probabilities are conditioned on priors obtained from the stochastic context-free grammar:

The posterior probabilities are calculated using the inside-outside algorithm [16]. The structure returned by *pfold *is the structure with the maximum number of expected correctly predicted nested structures. The matrix of expectation values is defined by the following recursion relation:

with initialization conditions *E_i,i _*= *P_s_*(*i*) for all *i*, where the basepair probabilities *P_d _*and unpaired base probabilities *P_s _*are obtained from the inside-outside variables. The final structure returned to the user is obtained by backtracking in this matrix. The reader is advised to consult references [10] and [11] for more details on the *pfold *model and algorithm.

## Authors' contributions

ZS implemented *PPfold *and wrote the manuscript. BK advised programming design and helped with debugging the algorithm. MV provided practical help and code for integration into the CLC Workbenches. ZS and ESA carried out the tests of the algorithm. ESA and JK managed the funding for the project, evaluated the test results, provided feedback for design improvements and critically revised the manuscript. All authors read and approved the final manuscript.

## Supplementary Material

Additional file 1 **Animation of distribution of the SCFG calculations**. The animation demonstrates the asynchronous wavefront computational approach, and was created on the basis of actual runtime data during the folding of a sequence of 460 nt, on a 2-core machine. The animation is divided into three parts: the inside (red/green), outside (yellow/blue) and expectation (cyan/magenta) calculations. The first colour represents jobs that are ready to be executed (because all their dependencies are fulfilled), and are therefore placed in a queue. At any time during the animation, the two jobs that entered the queue earliest are under execution (not shown), as the execution happens in a threadpool corresponding to the available number of cores (here, 2). When a job is finished, it changes to the second colour, and any newly available jobs (with finished dependencies) are pushed onto the queue. Click here for file
